# Systematic Review of Health Economic Evaluation Studies Developed in Brazil from 1980 to 2013

**DOI:** 10.3389/fpubh.2018.00052

**Published:** 2018-02-28

**Authors:** Tassia Cristina Decimoni, Roseli Leandro, Luciana Martins Rozman, Dawn Craig, Cynthia P. Iglesias, Hillegonda Maria Dutilh Novaes, Patrícia Coelho de Soárez

**Affiliations:** ^1^Department of Preventive Medicine, Faculty of Medicine, University of São Paulo, São Paulo, Brazil; ^2^Institute of Health and Society, Newcastle University, Newcastle, United Kingdom; ^3^Center for Health Economics, University of York, York, United Kingdom

**Keywords:** economic evaluation, cost-effectiveness, Brazil, cost-benefit analysis, health technology assessment

## Abstract

**Background:**

Brazil has sought to use economic evaluation to support healthcare decision-making processes. While a number of health economic evaluations (HEEs) have been conducted, no study has systematically reviewed the quality of Brazilian HEE. The objective of this systematic review was to provide an overview regarding the state of HEE research and to evaluate the number, characteristics, and quality of reporting of published HEE studies conducted in a Brazilian setting.

**Methods:**

We systematically searched electronic databases (MEDLINE, EMBASE, Latin American, and Caribbean Literature on Health Sciences Database, Scientific Electronic Library Online, NHS Economic Evaluation Database, health technology assessment Database, Bireme, and *Biblioteca Virtual em Saúde Economia da Saúde*); citation indexes (SCOPUS, Web of Science), and *Sistema de Informação da Rede Brasileira de Avaliação de Tecnologia em Saúde*. Partial and full HEEs published between 1980 and 2013 that referred to a Brazilian setting were considered for inclusion.

**Results:**

In total, 535 studies were included in the review, 36.8% of these were considered to be full HEE. The category of healthcare technologies more frequently assessed were procedures (34.8%) and drugs (28.8%) which main objective was treatment (72.1%). Forty-four percent of the studies reported their funding source and 36% reported a conflict of interest. Overall, the full HEE quality of reporting was satisfactory. But some items were generally poorly reported and significant improvement is required: (1) methods used to estimate healthcare resource use quantities and unit costs, (2) methods used to estimate utility values, (3) sources of funding, and (4) conflicts of interest.

**Conclusion:**

A steady number of HEE have been published in Brazil since 1980. To improve their contribution to inform national healthcare policy efforts need to be made to enhance the quality of reporting of HEEs and promote improvements in the way HEEs are designed, implemented (i.e., using sound methods for HEEs) and reported.

## Introduction

Brazil is an upper middle-income country with a population of 200 million citizens, largely urban (85%). It is a federative republic with 26 states, a federal district, and 5,564 municipalities. The 1998 Brazilian Constitution offered the right to health for all citizens, and created the Unified Health System (SUS): a public system directed at provision of universal, comprehensive, collective, and individual healthcare. The major SUS funders are federal, states, and cities governments, through taxes and social contributions. Public and private providers deliver services which are free at the point of delivery. The private sector covers approximately 25% of the population (48 million people), and dominated by an emergent health insurance market (1).

In 2014, Brazil had a gross domestic product (GDP) per capita of US$15,200, and approximately 9% of its GDP was spent on healthcare. The health expenditure per capita is US$1,109, and 46% of this is funded by public sources (2). The SUS public health financing is considered insufficient, and the equilibrium between public and private systems is challenging with considerable funds flowing from public coffers to private providers (3).

Resource scarcity is a reality in the Brazilian health system. Due to this scarcity, efficient allocation of resources is essential. Economic evaluation methods in healthcare have evolved as an important tool to assess the costs and benefits of health technologies and help decision-makers inform efficient allocation.

Brazil has sought to use economic evaluation to support decision making for rational management of the health system. The Ministry of Health, through the Department of Science and Technology (DECIT), has fostered the development of economic evaluation studies. Since 2006, DECIT has collaborated with the Committee on Incorporation of Technologies of the Ministry of Health (CITEC), an health technology assessment (HTA) body responsible for evaluating the incorporation of new technologies by SUS (4). In 2011, CITEC was replaced by the National Incorporation of Technologies in SUS, CONITEC, introduced the requirement for health economic evaluation (HEE) studies either to help inform policy recommendations for the adoption of new technologies or to review policy recommendations made by SUS (5, 6). The first Brazilian guideline for HEE studies was published in 2009, but the concept of a reference case has not been prescriptively adopted in Brazil yet. The revised HEE Brazilian guideline (7) issued by the Ministry of Health in 2014, presents some recommendations that broadly agree on many methodological specifications of the National Institute for Health and Care Excellence reference case.

In recent decades, a large amount of local HEE studies have been published. There is a strong evidence of the upward stream of blossoming in HEE publications and its acceleration (8). The evolution of scientific literature in health economics published in Brazil between 1986 and 2007 has been evaluated and reported in the published literature (9–11). Recently, Brazil appeared among the top 15 countries in HE research, accounting for 1.7% of identified records, and has been identified as the South American country that published the largest number of HEE studies (8, 12, 13).

Systematic reviews of country-specific HEE studies were conducted earlier in other countries including developed countries, Latin America, Asian, and African countries (14–28).

While a number of HEE have been conducted in Brazil, no study has systematically reviewed the quality of Brazilian HEE. The objective of this systematic review was to assess the state of the HEE research capacity development in Brazil and the ability to conduct good quality HEE. Specifically, this review evaluated the number, characteristics, and quality of reporting of published economic studies in a Brazilian setting.

## Materials and Methods

This study followed the guidelines for systematic review of HEE studies published by the Centre for Reviews and Dissemination (CRD) and the preferred reporting items for systematic reviews and meta-analyses statement (29, 30). The protocol is available from the authors on request.

### Systematic Search and Identification of Relevant Studies

A broad and exhaustive strategy search was formulated in order to identify all relevant studies published between January 1980 and December 2013. We systematically searched the following electronic data bases: MEDLINE (*via* PubMed), EMBASE, Latin American, and Caribbean Literature on Health Sciences Database, Scientific Electronic Library Online, NHS Economic Evaluation Database, HTA Database (CRD), Bireme, and *Biblioteca Virtual em Saúde Economia da Saúde*; citation indexes: SCOPUS, Web of Science, and the *Sistema de informação da Rede Brasileira de Avaliação Tecnologia e Saúde* (SISREBRATS). We also performed manual searches from the reference lists of included articles, and all issues of the Brazilian Journal of Health Economics (BJHE), a non-indexed journal in the previously mentioned databases in 2013.

The search strategy was reviewed by a librarian specialist and combined subject headings (MeSH and EMTREE) and free text terms (“Health Economics” OR “Economics, Hospital” OR “Economics, Medical” OR “Economics, Nursing” OR “Economics, Pharmaceutical” OR “Economics” OR “costs and cost analysis” OR “Cost” OR “Cost savings” OR “Cost of illness” OR “Analyses, Cost-Benefit” OR “Analysis, Cost-Benefit” OR “Cost-Benefit Analyses” OR “Cost Benefit Analysis” OR “Analyses, Cost Benefit” OR “Analysis, Cost Benefit” OR “Cost Benefit Analyses” OR “cost Effectiveness” OR “Effectiveness, Cost” OR “cost effectiveness analysis” OR “cost-Benefit Date” OR “cost Benefit Date” OR “Date, Cost-Benefit” OR “cost Benefit” OR “Benefits and Costs” OR “Costs and Benefits”) for “economic/cost” concept with subject headings (MeSH and EMTREE) and free text terms (“Brazil” OR “Brazilian” OR “Brazi*”) for “Brazil” concept. Keywords were matched to database specific indexing terms, taking into account the change in the indexing or classification of economic studies in different databases.

### Eligibility: Selection Criteria

Articles were included if they were partial or full HEE according to internationally recognized criteria (31, 32), referred to the Brazilian setting, and at least one of the authors was Brazilian and affiliated to a Brazilian institution. Multicenter studies, where Brazil was one of the participating countries, as well as studies conducted on Brazil by foreign authors, were excluded.

Studies were considered partial HEE if they examined only costs (cost description), described costs of a particular disease to society (cost of illness), described costs and outcomes of a single service or program (cost-outcome description), described financial consequences of technology adoption [budget impact analysis (BIA)] or compared only costs of two or more interventions (cost analysis). Studies were considered full HEE if they compared costs and consequences of two or more healthcare interventions alternatives, including cost-consequences analysis (CCA), cost-minimization analysis (CMA), cost-effectiveness analysis (CEA), cost-utility analysis (CUA), and cost-benefit analysis (CBA).

Abstracts, editorials, letters, posters and congress communications, methodological, discussion and review articles, and economic evaluation of other than health technologies (for example, environment) were excluded.

The titles and abstracts of identified citations were screened for relevance independently by two reviewers (Tassia Cristina Decimoni and Roseli Leandro). Disagreements were resolved through discussion or through consultation with a third reviewer (Patrícia Coelho de Soárez). Full texts of selected and those for which inclusion was in doubt were retrieved and independently screened by both reviewers.

### Data Extraction

Two reviewers (Tassia Cristina DecimoniTCD and Roseli Leandro) independently extracted data from each of the included studies on year and journal of publication, economic evaluation type, category of technology assessed (drugs, vaccines, equipment, clinical, surgical and diagnostic procedures, public health and health promotion programs), objective of the technology assessed (treatment, prevention, screening, and diagnosis), health problem studied (International statistical classification of diseases and related health problems, 10th revision, ICD-10) (33), first author affiliation (academy, government, research institutes, health organization, consulting, pharmaceuticals or equipment industry, international body), region of the first author, source of funding (research funding agencies, government, consulting, pharmaceuticals, or equipment industry), and authors’ conflict of interest. Conflict of interest was defined according to Valachis et al. (34) who argued that an author may need to declare having a conflict of interest in if she/he has received remuneration in payment or in kind (e.g., stocks or shares) from the manufacturer as a result of any of the following: research support or employment contract (salary, equipment, supply, reimbursement for participation in symposia, and other expenses), or consulting services.

In addition to the above, the standardized extraction form used also contained 17 questions from a systematic review of quality assessment tools (35), and the Consolidated Health Economic Evaluation Reporting Standards (CHEERS) instrument (36). These questions were intended to assess the quality of reporting of full HEE. Quality was defined as the extent to which a study complied and reported items included in the quality assessment tool and CHEERS checklist mentioned above (35, 36). The quality of the sources of evidence used in the studies was assessed with the hierarchy proposed by Coyle and Lee (37, 38). Where data sources ranked 1 are considered to be the most appropriate source (highest quality), and those assigned a rank of 6 are considered the least appropriate (lowest quality) (39) (Table 1). Disagreements on the extracted data were resolved through discussion or through consultation with a third reviewer (Patrícia Coelho de Soárez).

**Table 1:** Hierarchies of data sources for health economic evaluation studies modified from Coyle and Lee (37–39).

Rank	Data components
**Clinical effect sizes, adverse events, and complications**	

1+	Meta-analysis of RCTs with direct comparison between comparator therapies measuring final outcomes
1	Single RCT with direct comparison between comparator therapies measuring final outcomes
2+	Meta-analysis or RCTs with direct comparison between comparator therapies measuring the surrogate outcomesMeta-analysis or placebo-controlled RCTs with similar trial populations, measuring final outcomes for each individual therapy
2	Single RCT with direct comparison between comparator therapies measuring the surrogate outcomesSingle placebo-controlled RCTs with similar trial populations, measuring final outcomes for each individual therapy
3+	Meta-analysis or placebo-controlled RCTs with similar trial populations, measuring the surrogate outcomes
3	Single placebo-controlled RCTs with similar trial populations, measuring the surrogate outcomes for each individual therapy
4	Case control or cohort studies
5	Nom-analytic studies, for example, case reports, case series
6	Expert opinion

**Resource use**	

1	Prospective data collection or analysis of reliable data for specific study
2	Recently published results of prospective data collection or recent analysis of reliable administrative data—same jurisdiction
3	Unsourced data from previous economic evaluation—same jurisdiction
4	Recently published results of prospective data collection or recent analysis of reliable administrative data—different jurisdiction
5	Unsourced data from previous economic evaluation—different jurisdiction
6	Expert opinion

**Costs**	

1	Cost calculations based on reliable databases or data sources conducted for specific study—same jurisdiction
2	Recently published cost calculations based on reliable databases or data sources—same jurisdiction
3	Unsourced data from previous economic evaluation—same jurisdiction
4	Recently published cost calculations based on reliable databases or data sources—different jurisdiction
5	Unsourced data from previous economic evaluation—different jurisdiction
6	Expert opinion

**Utilities (if applicable)**	

1	Direct utility assessment for the specific study from a sample either: (a) of the general population (b) with knowledge of the disease(s) of interest (c) of patients with the disease(s) of interest Indirect utility assessment from specific study from patient sample with the disease(s) of interest, using a tool validated for the patient population
2	Indirect utility assessment from a patient sample with the disease(s) of interest, using a tool not validated for the patient population
3	Direct utility assessment from previous study from a sample either: (a) of the general population (b) with knowledge of the disease(s) of interest (c) of patients with the disease(s) of interest Indirect utility assessment from previous study from patient sample with the disease(s) of interest, using a tool validated for the patient population
4	Unsourced utility data from previous study—method of elicitation unknown
5	Patient preference values obtained from a visual analog scale
6	Delphi panels, expert opinion

*RCT, randomized control trial*.

### Data Summary

Data were summarized using qualitative narrative synthesis. The study characteristics are summarized in figures and summary tables.

### Data Analysis

Data analysis was performed with descriptive statistics such as absolute frequencies (raw counts) for each category of the discrete variable, relative frequencies (proportions or percentages of the total number of observations), along with analytic statistics that included Pearson correlations to investigate the relationship between the quality of reporting of the full HEE and the publication time period (1980–2005, 2006–2009, and 2010–2013), conflict of interest and source of funding. The publication time periods were chosen because they represent three different stages of the HTA in Brazil (1980–2005: before the establishment of the General Coordination Office for HTA; 2006–2009: establishment of CITEC; 2010–2013; after the publication of the Brazilian guideline for HEE studies). Linear regression models were used to evaluate changes in study characteristics over time. Data analyses were conducted using STATA/SE version 12.1 (Stata Corp, College Station, USA). An alpha level of 5% was used for statistical significance (*P* ≤ 0.05).

## Results

### Literature Search

**Figure 1:**
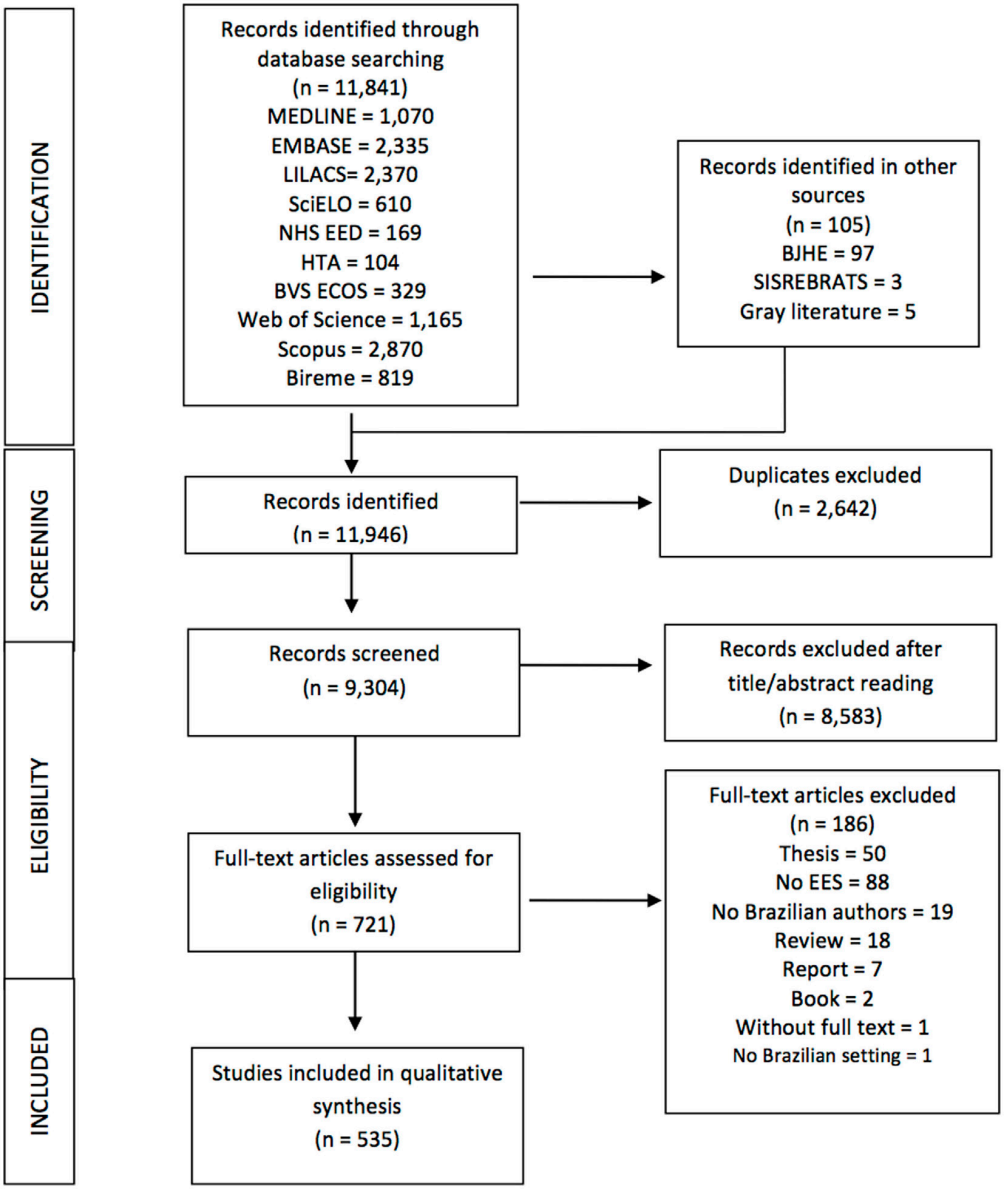
Flow diagram of health economic evaluation studies in Brazil, 1980–2013.

In total 11,841 records were identified from database searches, and 105 further articles were identified through hand-searching in BJHE, SISREBRATS, and other sources. Figure 1 depicts a flow diagram with full details of searches output, and reasons for inclusion/exclusion. We identified 9,304 non-duplicate citations, of which 721 were recognized as potentially relevant and full papers were retrieved. Out of the 721 studies 186 of them were excluded, reasons for exclusion included: thesis (50 studies), not HEE (88 studies), no Brazilian author (19 studies), reviews (18), and other (11 studies), see Figure 1 for detailed description. Scientific papers derived from excluded thesis and reports were included.

### Study Characteristics

**Figure 2:**
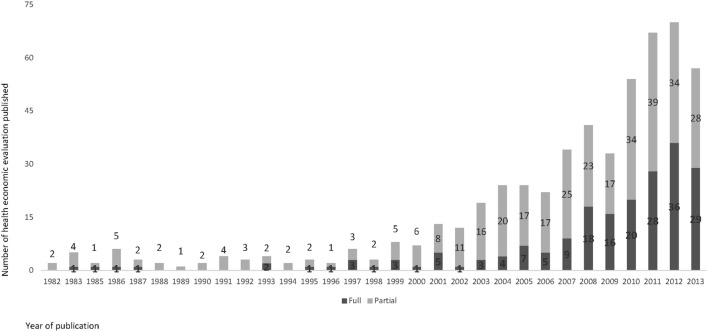
Number of health economic evaluation studies in Brazil, by type, 1980–2013.

The publication of HEE studies in Brazil started in the 1980s. Since then, there has been an upward trend with a slight increase at the end of the 1990s, and a sharp increase in 2007 with the publication of 356 (67%, 356/535) articles (Figure 2). A total of 535 studies were identified as suitable for inclusion in this review and their characteristics are described in Table 2.

**Table 2:** Characteristics of health economic evaluation (HEE), according time period, Brazil, 1980–2013.

Characteristics	1980–1989	1990–1999	2000–2004	2005–2009	2010–2013	Total	*P*-value
**Type of HEE**							

**Partial**	***N*(%)**	***N*(%)**	***N*(%)**	***N*(%)**	***N*(%)**	***N*(%)**	

Cost description	11 (4.9)	15 (6.7)	37 (16.6)	64 (28.7)	96 (43.0)	223 (100)	0.133
Cost analysis	5 (4.6)	11 (10.1)	23 (21.1)	35 (32.1)	35 (32.1)	109 (100)	0.155
Cost-outcome description	1 (25.0)	–	1 (25.0)	–	2 (50.0)	4 (100)	0.472
Cost analysis and BIA	–	–	–	–	2 (100)	2 (100)	0.183

Total	17 (5.0)	26 (7.7)	61 (18.0)	99 (29.3)	135 (39.9)	338 (100)	0.168

**Full**	***N*(%)**	***N*(%)**	***N*(%)**	***N*(%)**	***N*(%)**	***N*(%)**	

Cost-effectiveness analysis	2 (2.6)	–	4 (5.2)	26 (33.8)	45 (58.4)	77 (100)	0.150
Cost-consequence analysis	–	6 (15.0)	8 (20.0)	12 (30.0)	14 (35.0)	40 (100)	0.0001
CEA and CUA	–	1 (4.8)	–	5 (23.8)	15 (71.4)	21 (100)	0.150
Cost–utility analysis	–	–	–	3 (17.6)	14 (82.4)	17 (100)	0.028
Cost-minimization analysis	2 (14.3)	1 (7.1)	1 (7.1)	4 (28.6)	6 (42.9)	14 (100)	0.03
Cost-benefit analysis	–	2 (22.2)	–	1 (11.1)	6 (66.7)	9 (100)	0.133
More than one	–	1 (5.3)	1 (5.3)	4 (21.1)	13 (68.4)	19 (100)	0.355

Total	4 (2.0)	11 (5.6)	14 (7.1)	55 (27.9)	113 (57.4)	197 (100)	0.995

**Type of technology**	***N*(%)**	***N*(%)**	***N*(%)**	***N*(%)**	***N*(%)**	***N*(%)**	

Procedures	8 (4.3)	20 (10.8)	35 (18.8)	50 (26.9)	73 (39.2)	186 (100)	0.002
Medications	8 (5.2)	4 (2.6)	14 (9.1)	50 (32.5)	78 (50.6)	154 (100)	0.063
Procedures and medications	3 (4.6)	1 (1.5)	8 (12.3)	17 (26.2)	36 (55.4)	65 (100)	0.132
Public health and health promotion programs	–	4 (8.5)	4 (8.5)	14 (29.8)	25 (53.2)	47 (100)	0.176
Devices	1 (4.3)	2 (8.7)	5 (21.7)	7 (30.4)	8 (34.8)	23 (100)	0.287
Vaccines	–	3 (14.3)	2 (9.5)	8 (38.1)	8 (38.1)	21 (100)	0.777
Procedure, medications, and devices	–	1 (8.3)	1 (8.3)	4 (33.3)	6 (50.0)	12 (100)	0.564
Equipment	–	1 (33.3)	1 (33.3)	–	1 (33.3)	3 (100)	0.251
Other	1 (4.2)	1 (4.2)	5 (20.8)	4 (16.7)	13 (54.2)	24 (100)	0.795

**Objective**	***N*(%)**	***N*(%)**	***N*(%)**	***N*(%)**	***N*(%)**	***N*(%)**	

Treatment	18 (4.7)	22 (5.7)	55 (14.2)	115 (29.8)	176 (45.6)	386 (100)	0.796
Prevention	–	5 (10.9)	4 (8.7)	16 (34.8)	21 (45.7)	46 (100)	0.588
Diagnostic and treatment	–	–	8 (25.0)	6 (18.8)	18 (56.3)	32 (100)	0.197
Diagnostic	1 (3.2)	4 (12.9)	2 (6.5)	9 (29.0)	15 (48.4)	31 (100)	0.989
Screening	–	2 (15.4)	–	4 (30.8)	7 (53.8)	13 (100)	0.591
Prevention and treatment	–	2 (40.0)	1 (20.0)	1 (20.0)	1 (20.0)	5 (100)	0.079
Screening, diagnostic, and treatment	–	–	1 (100)	–	–	1 (100)	0.336
Other	2 (9.5)	2 (9.5)	4 (19.0)	3 (14.3)	10 (47.6)	21 (100)	0.278

Total	21 (3.9)	37 (6.9)	75 (14.0)	154 (28.8)	248 (46.4)	535 (100)	0.734

*BIA, budget impact analysis; CEA, cost-effectiveness analysis; CUA, cost-utility analysis; more than one: 19 studies concurrently performed more than one type of analysis: 13 studies CEA and BIA; 1 study CMA and BIA; 1 study CUA and CBA; 1 study CEA and CBA; 1 study CMA and CBA; 1 study CEA, CUA, and BIA; 1 study CMA, CBA, and CCA*.

**Figure 3:**
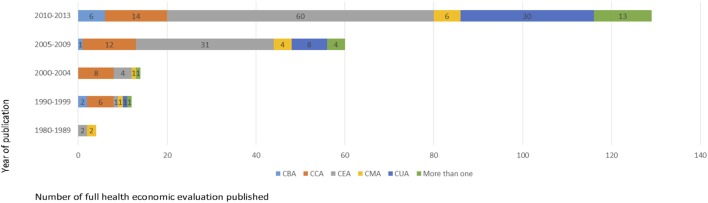
Full health economic evaluation published in Brazil, 1980–2013. More than one: cost-effectiveness analysis (CEA) and budget impact analysis (BIA); cost-minimization analysis (CMA) and BIA; CMA, cost-benefit analysis (CBA), and cost-consequences analysis (CCA); ACU and ACB; CMA and CBA; CEA, cost-utility analysis (CUA), and BIA.

According to internationally agreed classifications of HEE studies (31, 32), more than half of included studies were partial HEE (63.2%, 338/535). Of these, the majority (66%, 223/338) were cost description followed by cost analysis studies (32.2%, 109/338). Of the 197 full HEE, 39.1% (77/197) were CEA, 20.3% (40/197) were CCA, 11% (21/197) were CEA and CUA, 8.6% (17/197) were CUA, 7.1% (14/197) were CMA, and 4.6% (9/197) were CBA. Nine percent (19/197) of the studies concurrently performed more than one type of analysis. CMA evaluated mainly medications (corticosteroids, antihistamines, antibiotics, biologics, monoclonal antibodies, tyrosine kinase inhibitors, chemotherapeutics, anticoagulants, etc.). Prior to 2008, the majority of published evaluations were partial HEE. From 2008 onward, there was an increase in the number of full HEE, and the distribution of full and partial HEE studies became almost equivalent. An initial increase in the number of CEA studies has been followed by a sharp rise in the number of CUA, these have almost quadrupled in the last 3 years. The proportion of CUA studies increased from 17.6% between 2004 and 2009 to 82.4% between 2010 and 2013 (*P* = 0.028) (Table 2; Figure 3). Out of the 535 included studies, nearly half (*n* = 248, 46.3%) did not report the type of HEE study performed. Among these, 228 were partial and 20 were full HEE.

The review indicated an issue with the classification of study design in the identified HEE studies. According to international criteria (31, 32) of the 287 HEE that reported study type, 28.5% (82/287) had been classified incorrectly. Fifty-two (63.4%, 52/82) were partial analysis and 36.6% (30/82) full analysis. The most frequent misclassification among full HEE was to describe studies as CEA, where on investigation they were found to be CCA (53%, 16/30). Similarly, partial analyses described as cost analyses were cost descriptions (33%, 17/52). Some studies reported as CBA only performed cost analyses (29%, 15/52). Finally, some studies described as cost descriptions were cost analyses (15%, 8/52).

The categories of healthcare technologies that were most frequently assessed were procedures (34.8%, 186/535) and drugs (28.8%, 154/535). The proportion of studies that evaluate procedures increased from 4.3% during the 1980s to 39.2% between 2010 and 2013 (*P* = 0.002). Technologies assessed included treatment (72.1%, 386/535), prevention (8.6%, 46/535), and diagnostic and treatment (6%, 32/535).

The technologies evaluated in the studies were mainly related to the group of diseases of the Chapter I—certain infectious and parasitic diseases of ICD-10 (17.4%, 93/535), followed by Chapter IX—diseases of the circulatory system (12.9%, 69/535), Chapter II—neoplasms (10.3%, 55/535), and Chapter IV—endocrine, nutritional, and metabolic diseases (9.2%, 49/535).

### Studies by Authorship, Journal, Funding Source, and Conflict of Interest

**Figure 4:**
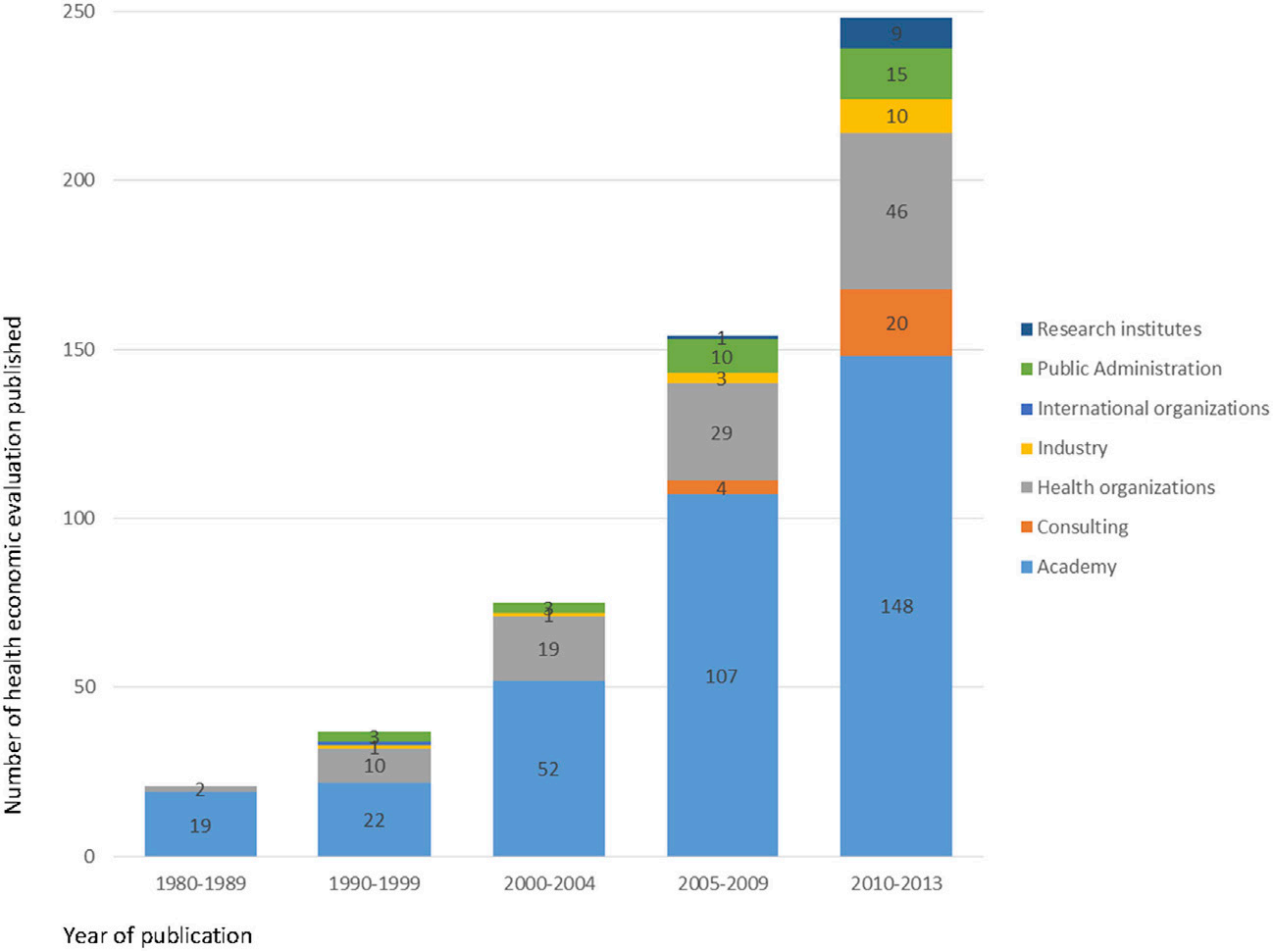
Number of health economic evaluation studies in Brazil, by first author affiliation, 1980–2013.

In most of the studies evaluated, the first authors were affiliated to academic institutions (65.1%, 348/535), followed by health organizations (19.8%, 106/535), public administration (5.8%, 31/535), consultancy firms (4.5%, 24/353), pharmaceuticals or equipment industry (2.8%, 15/535), research institutes (1.9%, 10/535), and international organizations (0.2%, 1/535). Although in the majority of publications, the first author was affiliated to an academic institution, there has been an increase of first authors affiliated to health organizations and consultancy firms (Figure 4).

The majority of Brazilian HEE studies were published in medical (55.5%, 297/535), and, public health (20%, 107/535) journals. Only 11.9% (64/535) were published in specialized health economics journals. Three hundred and eighty-eight studies (72.5%, 388/535) were published in Brazilian journals. Of those, 10.6% (41/388) were published in a non-indexed journal.

Regarding the geographical distribution of the first authors, southeast region stands out as a major producer of HEE (73.6%), followed by south (12.5%), and northeast (8.2%). The proportion of publications by regions remained constant during the study period. São Paulo and Rio de Janeiro were the Brazilian states more productive, 51.6 and 14.6%, respectively.

Two hundred and thirty-four studies (44%, 234/535) reported the funding source, among these, 12.4% (29/234) reported no funding and 87.6% (205/234) reported some funding source. Of these, 39% (80/205) were funded by research agencies, 32% (65/205) by industry, 15% (31/205) by the government, and 14% (29/205) had other or multiple funding sources.

Of the 535 studies included in the review, 36% (193/535) declared a conflict of interest, 82% (159/193) declared no conflict of interest. Of the 159 studies that declared no conflicts of interest, 13% (21/159) were considered (according to Valachis et al.) (34) to have a potential conflict of interest due to authors being industry or consultancy firm employees. Similarly, applying Valachis criteria to all 535 included studies, 84% (449/535) would be considered to not have a conflict of interest and 16% (86/535) could be considered to have a conflict of interest. Identified reasons for potential conflict of interest were: 49% (42/86) were developed by consultancy firms and industry; 45% (39/86) had at least one author contractually employed by the industry or funded by it; and 6% (5/86) were related to consultancy work.

**Figure 5:**
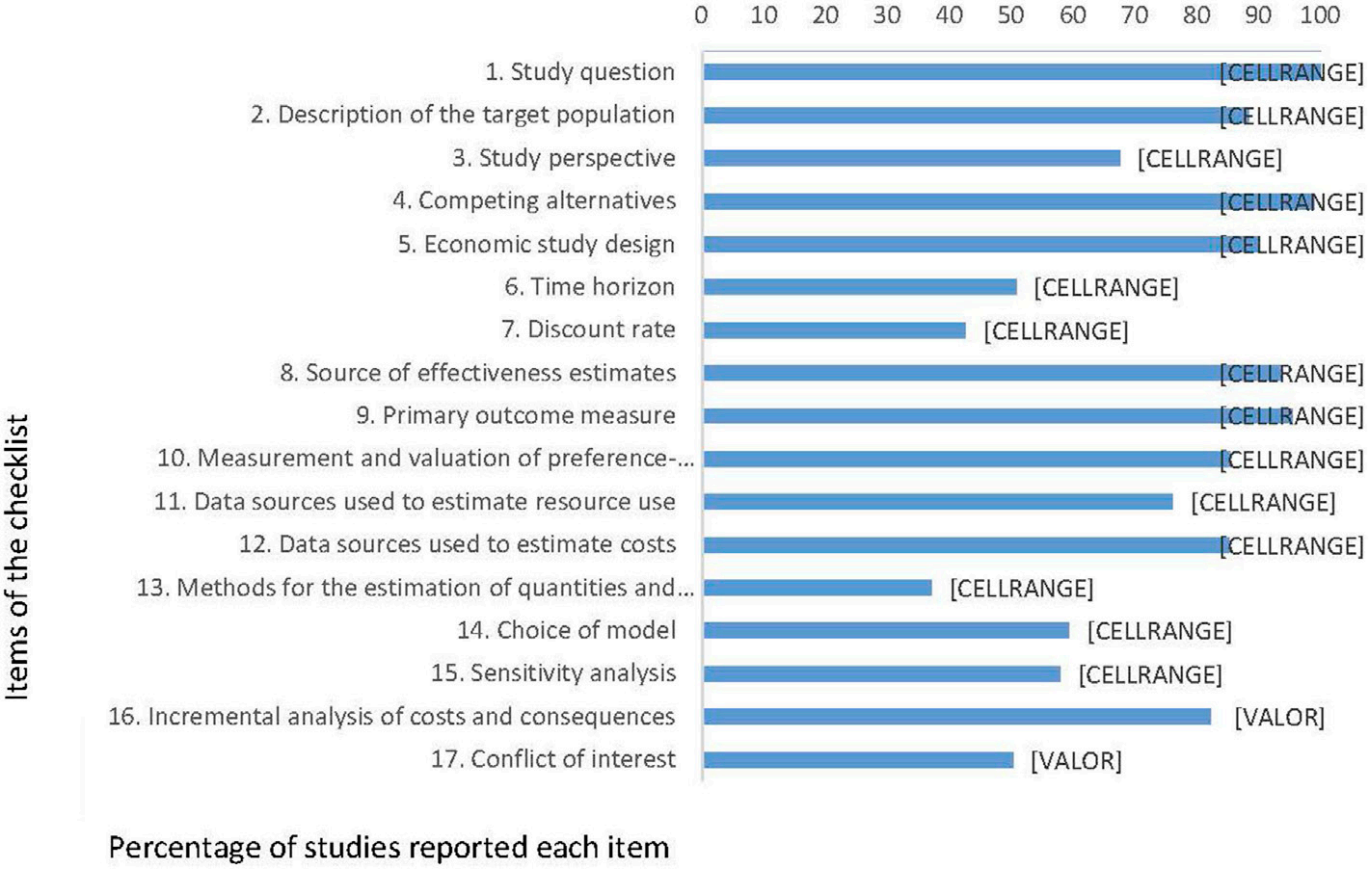
Percentage of studies complying with recommendations for reporting of full health economic evaluation (*n* = 197), Brazil, 1980–2013.

Compliance with international recommendations for good reporting in the 197 studies identified as full HEE are presented in Figure 5. Most studies complied with the following items clearly stated: the research question (100%), competing alternatives (99%), primary outcome measure (95%), source of effectiveness estimates (94%), type of model (92%), and economic study design (90%).

### Studies by Reporting Quality and Quality of the Sources of Evidence

Thirty-two percent (63/197) of the studies did not state a perspective for their analysis. Only 37% (73/197) described the methods for the estimation of total volume of healthcare resources used in a transparent manner (i.e., reporting quantities and prices/unit costs separately). Less than half (43%, 85/197) of the studies stated the discount rate applied. Other identified caveats associated with studies’ reporting were: failure to declare a conflict of interest, not stating a time horizon for the analysis, omitting to perform, and describe a sensitivity analysis. Of the 197 full HEE, 64.5% (127/197) were model-based economic evaluation. Of these, 92.1% (117/127) reported the type of model used: 41.9% (49/117) used Markov models, 34.2% (40/117) used decision trees, 6% (7/117) used Markov models with decision trees, 6% (7/117) dynamic models, and 12% (14/117) used other types of models.

The reporting quality association with the publication period was statistically significant (*P* < 0.001). The reporting quality increased progressively during the study period. Most recent periods showed better reporting quality. Studies published between 2010 and 2013 showed better reporting quality compared with those published between 2005 and 2009 and those published between 1980 and 2004. No association was found between the reporting quality and the study source of funding. Moreover, positive association was observed between the reporting quality and the variable conflict of interest. Studies with conflict of interest are associated with a better reporting quality (*P* < 0.001).

**Figure 6:**
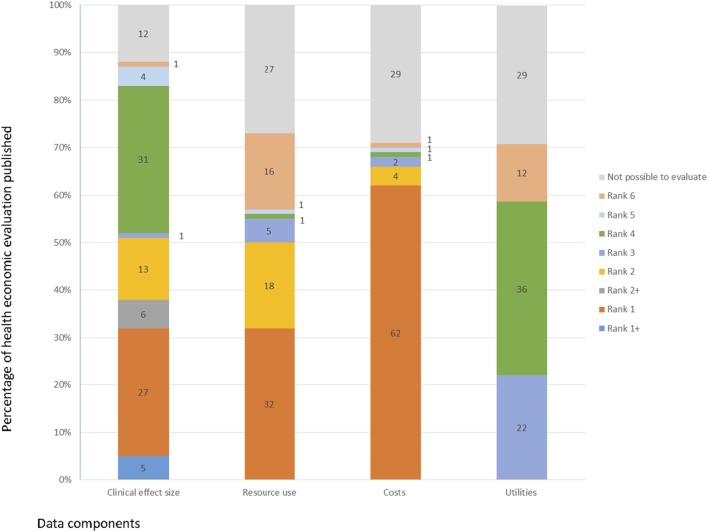
Quality of the sources of evidence used in the full health economic evaluation (*n* = 197), Brazil, 1980–2013.

The quality of the sources of evidence used in the studies performed in the Brazilian setting was analyzed in depth using the hierarchy proposed by Coyle and Lee (37–39). Figure 6 presents a graphical representation of results from this analysis. Our findings suggest that poorer quality of information was available for estimating utilities values than costs, resource use, and clinical effect size. No study directly (e.g., *via* a health state preference evaluation exercise) or indirectly (e.g., used utility values from an alternative patient sample but with the disease of interest) performed utility assessment. Twenty-two percent of the studies estimated utilities parameters from a previous study, 37% had data source or method of elicitation unknown, 12% was based on expert opinion, and 29% was not possible to evaluate. In contrast to utility value estimates data on costs, resource use and clinical effect size were mostly estimated using high-ranked evidence. For instance, more than 50% of the studies used cost information from sources where quality was ranked as 1+, 1, 2+, 2. Costs calculations were mainly based on reliable administrative database or data sources conducted for the specific study, and on recently published cost calculations based on reliable databases. Similarly, in 58% of the studies clinical effect sizes were estimated from meta-analysis or RCTs.

## Discussion

To the best of our knowledge, this the first systematic review describing the number, characteristics, and quality of reporting of HEE studies in Brazil.

The absolute number of all HEE published in Brazil between 1980 and 2013 (*n* = 535) is substantial. The volume of full HEE (*n* = 197) directly relevant to Brazilian settings is considerably higher than the number of HEE identified in other countries such as: Italy (*n* = 92), Colombia (*n* = 48), South Africa (*n* = 45), Thailand (*n* = 39), Korea (*n* = 33), Iran (*n* = 30), Vietnam (*n* = 26), China (*n* = 26), Saudi Arabia (*n* = 10), Bangladesh (*n* = 12), Nigeria (*n* = 10), and Zimbabwe (*n* = 3) (14, 15, 17, 19, 21, 24–28, 40). Conversely, Brazil still produces a lower number of HEE compared with high-income countries such as United Kingdom (*n* = 675), Canada (*n* = 300), German (*n* = 283), and Australia (*n* = 245), where economic evaluation is well established and formally used for regulating reimbursement policies for the adoption of new technologies (16, 20, 41).

BRICS (Brazil, Russia, India, and China) share in global wealth grew tremendously over past decade. In parallel to that, healthcare spending, health economic productivity, and research funding for health economics shifted toward low- and middle-income countries, especially in top emerging BRICS. These circumstances created drivers for further development of health economics in these countries (8, 42–44).

In recent years in Brazil, as in other LAC countries, there has been increased interest in incorporating HEEs as a formal tool to inform decision-making processes (12). This systematic review reveals a steady growing trend in the number of HEE studies being published in the last 6 years (2008–2013). Interestingly, this phenomenon is also observed in Colombia (26).

The steady growth in the economic evaluation literature relevant at national level in Brazil—the focus of this analysis—reached a peak in the year 2007. The reason for this may be multi-fold; firstly, from as early as 1980 Brazilian researchers, funders, and public health system users have successfully promoted the conduct and use of HEE studies. Secondly, internationally there was been an increased interest in HEE studies demonstrated by a continuous growth of published articles and books, as well as the creation of several new specialist journals in this field (45). A third crucially important factor is some recent initiatives of the Brazilian Ministry of Health such as CITEC and CONITEC establishments. The development of this formal structure for regulating reimbursement policies for the adoption of new technologies, provides a strong incentive to promote the implementation and publication of a growing number of HEE studies.

Findings from systematics reviews suggest that the increase in the number of HEE studies published internationally may be related to requirements to use information from these studies to inform reimbursement decisions (41).

As reported in earlier systematic reviews of HEE studies in Latin America (17, 26, 27), our findings indicated that CEA was the most prevalent (39.1%, 77/197) study type for full HEE conducted in Brazilian settings between 1980 and 2013. This might be explained by the relative simplicity of the CEA approach compared with CUA that requires developing robust methodology to value health state preferences.

Despite this, the current review found considerable growth in CUA for the Brazilian setting from 2005 onward (*P* = 0.028), in line with a growth in CUA observed in the international literature (41). The absolute number of CUA in Brazil (*n* = 40), however, is still small. This may, in part, be explained by the fact that CUA is more labor and resource intensive than CEA. In addition to this, the 2009 Brazilian HTA guideline gave equal weight to CEA and CUA (46). While CONITEC came into force in 2011 it did not update its methods guidelines to recommend the use of CUA. This is in contrast with current recommendations in a number of countries worldwide (e.g., Australia, Canada, Ireland, New Zealand, Scotland, Sweden, and United Kingdom) (47). CUA was indicated as the preferred type of study, only in the update of the Brazilian HEE guideline published in 2014 (48).

National Incorporation of Technologies in SUS has a formal requirement for BIA alongside a full HEE. In spite of this, the number of published BIA is still limited (*n* = 15), and most of the so-called BIA submitted to CONITEC are cost studies with 2–5-year annual costs for a specific cohort instead of a real estimate of the financial impact of a new intervention for the Brazilian healthcare system. These findings are consistent with a review of BIA studies (49).

Like other HEE in Latin America, Africa, and South/West Asia most studies focused on infectious disease (14, 21, 23, 36). The majority of these studies focused on vaccines, driven largely by increased investment on CEA and related activities by major global health players such as Bill & Melinda Gates Foundation, GAVI alliance, and the World Health Organization (48).

We found that 75.5% (297/535) of the studies were published in medical and public heath journals. In reviews of Spanish, Iran, German, and South African, HEE studies covering earlier years of publication, the majority of studies (77, 77, 79, and 88%, respectively) were also published in medical journals (13, 21, 24, 49). This high numbers may be related to lower publication standards in medical and public health journals when compared with specialized health economic journals.

Even though researchers prefer to publish their research in international journals, with higher impact factors, and a wider audience. Most of the studies (72.5%) were published in Brazilian journals. Among the 12% published in health economics specialized journals, 8% were published in a national health economic journal, which was not even indexed until 2013.

This may be related to publication requirements as editors of Brazilian journals have less stringent requirements to make use of international methodological guidelines as part of their peer reviewing processes. Consequently, articles with an inferior quality of reporting and equivocal methodological quality are published. Our data also suggest a need for improvements in the peer review process, especially among journals with limited experience publishing economic evaluations (50).

The majority (75%, 148/197) of identified full HEE studies were published between 2008 and 2013, and the reporting quality increased progressively during the study period (*P* < 0.001). Although overall quality of reporting was considered satisfactory, the review highlighted a number of issues associated with the reporting and methodological quality of the included HEE studies.

Two issues on quality of reporting deserve further attention. Firstly, reporting of funding source, 56%, 110 of the 197 full HEE studies identified here did not state their source of funding. This is in line with findings from reviews on the state of HEE studies conducted in South Africa (45%, 49/108) (24), Nigeria (55%, 24/44) (21), and Zimbabwe (62%, 16/26) (19). Secondly, and directly related to reporting source of funding is declaring potential for any conflicts of interest. Only 36% of the articles reported conflict of interest. As highlighted by Valachis et al. (34), conflicts of interest may be directly related to sources of funding. This is a phenomenon that has been researched by several authors (51–53), if studies are funded by the healthcare industry this could have a direct impact on the conclusion drawn. Industry-sponsored HEE are believed to be more likely to report incremental cost-effectiveness ratios that favor products manufactured by the sponsor. Missing details on these two crucial pieces of information described above may impact on the credibility and transparency of results from HEE studies.

Two final points are identified as requiring further consideration and recommendations for improvement, these are (1) methods for the estimation of resources quantities and unit costs and (2) methods for the estimation of utilities parameters. While the majority of studies provided a source for resource utilization and costs, they omitted details on the identification and quantification of categories of resources and estimation of unit costs. This is in contrast to HEE guidelines (54–56), which indicate that all the relevant quantities of resources should be measured in a correct and transparent manner, and reported separately from the prices (unit costs) of those resources. This lack of detailed reporting on the quantification of healthcare resources and methods used for their valuation limits the ability to replicate costing processes in future studies.

The final critical issue identified for discussion was the poor quality of information used for estimating utilities parameters. Authors’ often reported their main sources of information for the measurement and valuation of preference-based outcomes, these sources of evidence, however, were studies ranked as low quality. Many studies reported having used international data from previously published studies or expert opinion. This is in contrast with international guidelines on HEE which state that utility values obtained from other countries are, in general, not transferable because of cultural differences (57). Recently, in 2011, two of the most widely used generic preference-based utility instruments—EQ-5D and SF-6D—were cross-cultural adapted and validated, in addition societal preferences weights were estimated for the Brazilian population in 2013 (58–61). We expect that current efforts to estimate Brazilian utility weight “tariffs” will increase consistency in quality-adjusted life year calculations in future HEE studies.

One limitation of our study was that we critiqued the reporting, and not necessarily the actual manner that authors conducted their studies. However, this review was useful to assess the practice of HEE in the Brazilian setting.

## Conclusion

This review identified that an increasing number of HEE studies are being conducted and published in Brazil. Their reporting quality has increased progressively during the study period. Overall, the quality of these HEE studies is satisfactory, but we identified key areas where significant improvements could be made such as: reporting of funding source, conflict of interest, methods for the estimation of resources quantities and unit costs, methods and source of evidence to estimate utility parameters utilities parameters. Our findings can contribute to improve the way HEE studies are designed, implemented, and reported in Brazil.

## Consent for Publication

There are no any individual person’s data.

## Ethics Statement

There are no human participants involved.

## Author Contributions

All authors drafted the systematic review protocol. TD, RL, and PS conducted the search, selection of records, and data extraction. Quality appraisal was conducted by TD, LR, and PS. All authors have read and approved the final manuscript.

## Conflict of Interest Statement

The authors declare that the research was conducted in the absence of any commercial or financial relationships that could be construed as a potential conflict of interest.

## Abbreviations

HEEs, health economic evaluations; SUS, Unified Health System; GDP, gross domestic product; DECIT, Department of Science and Technology; CITEC, Committee on Incorporation of Technologies of the Ministry of Health; CONITEC, National Incorporation of Technologies in SUS; NICE, National Institute for Health and Care Excellence; CCA, cost-consequences analysis; CMA, cost-minimization analysis; CEA, cost-effectiveness analysis; CUA, cost-utility analysis; CBA, cost-benefit analysis; CHEERS, Consolidated Health Economic Evaluation Reporting Standards; HTA, health technology assessment; BJHE, Brazilian Journal of Health Economics; SISREBRATS, *Sistema de Informação da Rede Brasileira de Avaliação de Tecnologia em Saúde*; QALY, quality-adjusted life year.
